# Prostaglandin E1-Mediated Collateral Recruitment Is Delayed in a Neonatal Rat Stroke Model

**DOI:** 10.3390/ijms19102995

**Published:** 2018-09-30

**Authors:** Philippe Bonnin, Julien Pansiot, Olivier Baud, Christiane Charriaut-Marlangue

**Affiliations:** 1 Université Sorbonne Paris Cité, 75019 Paris, France; philippe.bonnin@aphp.fr (P.B.); Julien.Pansiot@inserm.fr (J.P.); Olivier.BAUD@hcuge.ch (O.B.); 2 Inserm U965, Université Denis Diderot, Physiologie Clinique—Explorations Fonctionnelles, Hôpital Lariboisière, 75010 Paris, France; 3 U1141 PROTECT, Inserm, Université Paris Diderot, Sorbonne Paris Cité, Hôpital Robert Debré, 48 Boulevard Sérurier, 75019 Paris, France; 4 Division of Neonatology and Pediatric Intensive Care, Children’s University Hospital of Geneva and University of Geneva, 1205 Geneva, Switzerland

**Keywords:** Neonatal ischemia, hemodynamic responses, ultrasound imaging, prostaglandins, thromboxane A synthase-1, Astrocytes

## Abstract

While arterial reflow after a stroke represents an important challenge for better outcomes, it is also very important that sudden recanalization does not produce local oxidative and nitrogen species, deleterious for the brain and more particularly the immature brain. Our objective was to determine whether a supply in prostaglandin (Pg) E1 (Alprostadil), via its action on arterial pressure, might progressively improve cerebral reperfusion in a neonatal stroke model. Arterial blood flow was measured using ultrasonography. Rate-limiting and Pg terminal synthesizing enzymes were evaluated using reverse-transcriptase polymerase chain reaction. Our data suggests that a supply in PgE1 might delay and improve the ipsilateral reperfusion by decreasing thromboxane A synthase-1 gene, the density of reactive astrocytes and lesion volume.

## 1. Introduction

Stroke in near and full-term babies represents a major cause of disabilities including cerebral palsy, mental retardation, visual and hearing problems, and epilepsy [1]. Hypothermia is the only therapy approved to attenuate brain injury in the newborn. However, this treatment is unfortunately only partially neuroprotective and can only be used to treat hypoxic-ischemic encephalopathy in full term infants. The best conceivable treatment for stroke, regardless supportive care is the restoration of blood flow (BF) to the affected region(s) as soon as possible because decreased cerebral BF (CBF) during the first 12 to 24 h indicates poor prognosis in infants with perinatal asphyxia [2].

Restoration of partial reperfusion in the penumbra by collateral circulation represents an important challenge; vasodilators can modulate reperfusion in preclinical ischemic models and are already used in neonatal intensive care units. Inhaled nitric oxide (iNO) mediated blood-flow redistribution during ischemia and lowered brain damage in the postnatal 7-day old (P7) rat [3]. Cerebral blood flow is regulated by neurons and astrocytes through the production and release of metabolites of arachidonic acid, which include prostaglandins (Pgs) and epoxyeicosatrienoic acids, and dilate nearby arterioles [4]. Using our classical model of ischemia-reperfusion [5,6], we recently reported that the juvenile P14 rat brains mostly respond to ischemia by a cyclo-oxygenase (Cox-2)- and microsomal PgE synthase-1-dependent prostaglandins production as demonstrated by reverse-transcriptase polymerase chain reaction [7].

While arterial recanalization is a prerequisite for better outcomes after stroke (in the immature and mature brain), we recently demonstrated that a progressive rather than a sudden restoration of arterial pressure and blood supply could be beneficial by avoiding the overwhelmed reperfusion and its subsequent local oxidative stress and nitrogen species production in the ischemic P14 rat [3,8]. The aim of this study was to evaluate whether treatment-using Pgs might improve collateral recruitment and brain damage after ischemia in the neonatal P7 rat. In contrast to the immediate vasodilation produced by inhaled NO [3], prostaglandin E1 (PgE1) is known to induce hypotension and to dilate peripheral vessels but not cerebral arteries [9].

## 2. Results

The data were pooled as no difference was found in the male and female pups.

### 2.1. Neonatal Ischemia Induces Thromboxane a Synthase-1 Gene Expression

**Figure 1:**
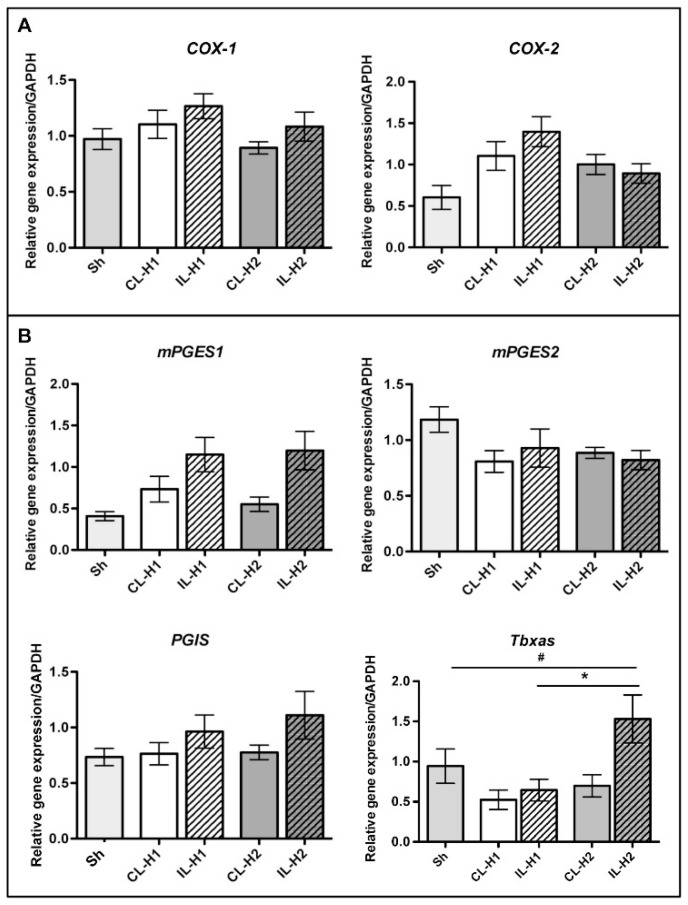
Delayed thromboxane A synthase-1 (*Tbxas*) up-regulation after ischemia. (**A**) Gene expression for *Cox-1* and *Cox-2* rate limiting enzymes in sham (light grey bar) and PBS-treated ischemic animals at 1 (H1) and 2 (H2) hours after reperfusion in the contralateral (CL) and ipsilateral side (IL). (**B**) Gene expression for terminal prostaglandins synthesizing enzymes in sham and PBS-treated animals. * *p* <0.05 H2 vs. H1; ^#^ *p* < 0.05 H2 vs. Sham. (*n* = 6 at each time point in each group, and in sham (Sh) animals). *mPGES1*: microsomal PgE synthase-1; *mPgES2*: microsomal PgE synthase-2; *PGIS*: prostacyclin synthase.

Ischemia in the P7 rat brain did not induce an increase in the rate-limiting cyclooxygenases (Cox-1 and Cox-2—Figure 1A) and principal terminal synthesizing enzymes specifying their nature (mPGES1, mPGES2 and PGIS—Figure 1B) at one and two h of recovery. However, we observed a significant increase in the thromboxane A synthase-1 gene expression (*Tbxas*) two hours after ischemia, as compared to sham (sh) animals (^#^ *p* < 0.05) and ischemic animals sacrificed one h after ischemia (* *p* < 0.05).

Rat pups then received during ischemia (50 min) either 4 injections (i.p., every five min between 30 and 45 min) of Aloprostadil (prostaglandin E1, PgE1) at a total dose of 20 µg/kg or PBS (same protocol).

### 2.2. PgE1 Induces Delayed Blood-Flow Redistribution in the Ipsilateral Side

**Figure 2:**
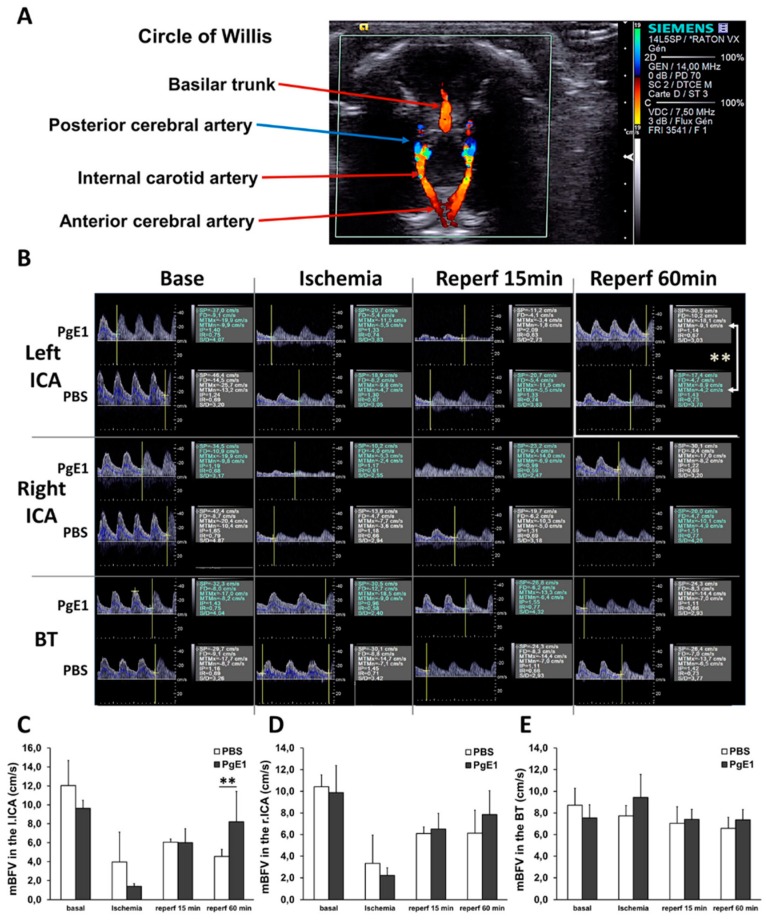
Blood-flow redistribution in the three great arteries of the circle of Willis after ischemia-reperfusion in P7 rats treated with PBS (*n* = 6) and/or prostaglandin E1 (PgE1) (*n* = 6). (**A**) Horizontal ultrasound cross-sectional imaging at the basis of the skull with color-Doppler mode showing the circle of Willis. The basilar trunk (BT) is revealed back, both intracranial internal carotid (ICA) and anterior cerebral arteries are revealed in red color at the front. The first branches of the ICAs, the posterior cerebral arteries are revealed in blue color (opposite flow-direction than in ICAs and BT). (**B**) After localization of the arteries, a pulsed-Doppler sample can be positioned on the different arteries for Doppler velocity waveforms recordings at the different steps of the protocol. (SP: systolic peak velocity; FD: end-diastolic velocity; MTMx: mean time maximal velocity; MTMn: mean time mean velocity; IP: index of pulsatility; IR: index of resistance; S/D systolic peak velocity/end-diastolic velocity. (**C**,**D**) is the mean blood flow velocities (mBFV) in the left and right ICAs decreased during ischemia; mBFVs recorded at 60 min of reperfusion remained lower than that measured in basal conditions in PBS-treated animals. In contrast, mBFVs returned to basal values in PgE1-treated rat pups (** *p* = 0.006, PgE1 vs. PBS). (**E**) mBFVs recorded in the BT showing no difference between PBS- and PgE1-treated animals. ** *p* < 0.01 PBS vs. PgE1.

The two groups of animals displayed similar mean blood-flow velocity (mBFV) under basal conditions in the three great arteries of the circle of Willis (Figure 2). At the end of ischemia, when both common carotid arteries (CCAs) were occluded, mBFV in the basilar trunk (BT) did not differ from that measured under basal conditions for the two groups (Figure 2E). We observed (for the two groups) residual mBFV in both intracranial carotid arteries (ICAs, Figure 2C,D), despite no visible flow in the CCAs, illustrative of collateral recruitment from the BT to the hemispheres (1) through the circle of Willis (BT to posterior communicant arteries, posterior cerebral arteries, then ICAs), or (2) through the cortical anastomoses that extend from the vascular supplies of the posterior and/or anterior cerebral arteries towards the vascular supply of the occluded left MCA. At early reperfusion (15 min), no difference in mBFV in the ICA(s) was observed between the two groups. In contrast, one h after reperfusion, mBFVs were restored (to the basal level) in the left ICA (*p* < 0.01 vs. PBS) only in PgE1-treated animals, whereas mBFVs remained low in the PBS group (Figure 2C). No significant variation in heart rates was detected between groups throughout the procedure.

### 2.3. PgE1 Reduces Thromboxane a Synthase-1 Expression and Lesion Size

**Figure 3:**
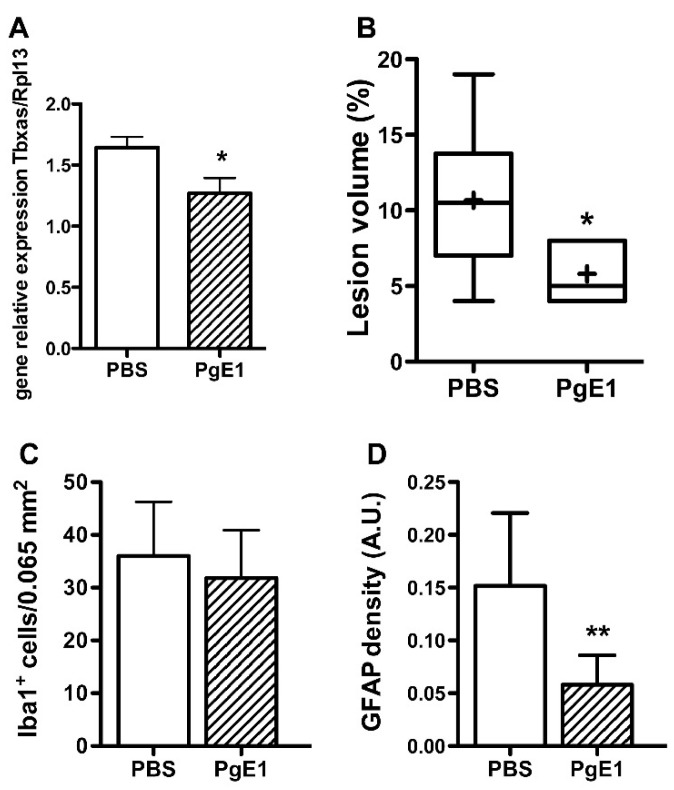
PgE1 induces better outcomes in neonatal ischemia at 48 h after ischemia-reperfusion. (**A**) Thromboxane A synthase-1 gene expression 2 h after ischemia-reperfusion in PBS- and PgE1-treated animals (*n* = 6 per group). (**B**) Infarct volumes in PBS- (*n* = 6) and PgE1-treated (*n* = 6) animals. The + indicates the mean. (**C**) Number of microglial cells and (**D**) GFAP density (astrocyte density) in the penumbra (*n* = 6 per group). * *p* < 0.05 vs. PBS; ** *p* < 0.01 vs. PBS.

The expression of tbxas gene was found reduced two hours after ischemia-reperfusion in PgE1-treated animals (*p* < 0.05 vs. PBS—Figure 3A). Forty-eight hours after ischemia-reperfusion, pale zones were observed on cresyl violet-stained sections (from Bregma 2.76 to −4.80 mm, Figure S2A) given a mean infarct volume of 10.7 ± 5.1% in PBS- and 5.8 ± 2.3% in PgE1-treated animals, respectively (Figure 3B). The number of Iba-1^+^ microglia was not different between the two groups of animals (Figure 3C and Figure S2B). In contrast, the density of GFAP (marker of reactive astrocytes) was reduced in PgE1-treated animals as compared to PBS-treated animals (*p* < 0.01, Figure 3D and Figure S2C).

## 3. Discussion

The present study shows that a supply in PgE1 improved blood-flow redistribution during reflow after ischemia in the neonatal P7, and reduced the size of the lesion. Increased collateral recruitment can be obtained by the administration of drugs to increase vasodilation [3,10] or by facilitating the synthesis of vasodilators [7] leading to the rerouting of BF through the numerous native anastomoses in the cerebral vascular network. We had also previously demonstrated that an increase in collateral supply induced by exogenous NO-donors is beneficial during ischemia but deleterious during reflow due to an elevation in oxidative stress and nitrogen species synthesis [3]. We here observed that PgE1 induced moderate blood-flow redistribution during reperfusion. This collateral recruitment is delayed compared to the drug administration (observed one h after reperfusion), and although it is moderate (reaching nearly the basal level), it improves blood circulation and oxygenation to the ischemic tissue in the ipsilateral hemisphere, most likely in the penumbra. However, we cannot exclude that the mechanisms involved in PgE1 supply could be more complex as increasing evidences suggest a cross talk between NO and prostaglandins biosynthetic pathways [11], but still remains to be better elucidated, namely during brain development.

We previously reported that arterial reflow in the P7 rat was slow and gradual in the carotid arteries [12], in contrast to that measured in the P14 rat with an immediate return to basal values [13], and to that reported in the adult brain with an hyperemic phase followed by an hypoperfusion [14]. Furthermore, we also reported that controlled arterial reflow (by progressive rerouting of arterial flow) induced better outcomes [8]. Thus, reperfusion is an important prerequisite to reduce brain damage, however this reperfusion should be moderate to avoid the uncontrolled reperfusion phase. Additional studies in rodents are needed to define the optimal rate of reperfusion.

Our study shows that a supply in PgE1 via a decrease in Tbxas formation reduced inflammatory responses via a reduction in the density of reactive astrocytes. Astrocytes and their endfoot processes envelop blood vessels and control blood flow via the production and release of metabolites of arachidonic acid (AA) including Pgs, prostacyclin, and thromboxanes [4]. One of the potentials signaling pathways could be a direct interaction between the supply in PgE1 and astrocytes in the neurovascular coupling. We cannot, however, exclude that the supply in PgE1 inhibits platelet aggregation, although platelet reactivity in term and preterm neonatal infants is often considered diminished or attenuated compared to adult.

In conclusion, a supply of either iNO or prostaglandins (this study) during ischemia plays a critical role in outcomes because early cerebral perfusion inversely correlates with the extent of the lesion in the neonatal rat brain [6]. Furthermore, and in contrast with iNO, PgE1 induced a delayed and moderate collateral recruitment during reflow thus avoiding excessive reperfusion and reducing brain exposure to local oxidative stress and nitrogen species synthesis extremely deleterious to the immature brain [15].

## 4. Materials and Methods

### 4.1. Neonatal Ischemia-Reperfusion

All experiments were carried out (license A75-19-01, Approval Date (15 January 2018), by the French Department of Agriculture in accordance with the European Committee’s Council Directive and performed to comply with the ARRIVE (Animal Research: Reporting In Vivo Experiments) guidelines. Ischemia was induced in Wistar P7 rat pups (16–18 g, *n* = 36; Janvier, Le Genest St-Isle, France; both sexes; 15 males and 21 females). Briefly, thermoregulated (37.0 ± 0.5 ℃) and anesthetized pups (induction 1.5%; maintenance 1% isoflurane in air) were exposed to left middle cerebral artery electocoagulation (pMCAo) combined with a transient (50 min) and concomitant occlusion of both common carotid arteries (CCA) [5,6]. Rat pups were sacrificed at one and two h (for gene expression) and at 48 h (lesion volume and immunohistochemistry) after reperfusion. Sham animals were also investigated and sacrificed two h after ischemia (for gene expression). No animal died throughout the experimental procedure and all animals were included in this study (Figure S1).

All experiments were performed in a blinded and randomized manner.

### 4.2. Drug Treatment

Animals were randomly assigned to PgE1 (Alprostadil Intsel Chimos 0.5 mg/mL; *n* = 24) or vehicle (PBS; *n* = 12). Prostine was four times injected during ischemia every 5 min (at 30, 35, 40 and 45 min, i.p.) given a total dose of 20 µg/kg (Figure S1), as intravenous perfusion was not easy to perform in neonatal rat pups. The dose was chosen according to that used in newborns. Pg E1 has several pharmacological effects, including vasodilation, inhibition of platelet aggregation, membrane stabilization and anti-inflammation, and is classically used in the treatment of peripheral arterial occlusive disease in infants [16].

### 4.3. Sample Size Calculation

Assuming α β risk of 0.2 and an α risk of 0.05, it was estimated (using the BiostaTGV software (https://marne.u707.jussieu.fr/biostatgv)) that six animals in each group were needed to observe a significant difference in gene expression and US imaging as previously reported [7,8].

### 4.4. Quantitative Polymerase Chain Reaction Analysis

Animals (sham, PBS-treated and Prostine-treated) were sacrificed at one and two h after reperfusion (*n* = 6 per group at each of the two time points), and the cortical tissue (all along the MCA) harvested. Total RNA was extracted using RNeasy lipid tissue mini kit (Qiagen, Courtaboeuf, France). RNA quantity and quality were assessed by spectrophotometry with the Nanodrop^TM^ apparatus (Thermoscientific, Wilmington, DE, USA). Total RNA (1–2 µg) was subjected to reverse transcription using the iScript^TM^ cDNA synthesis kit (Bio-Rad, Marnes-la-Coquette, France). RT-qPCR was performed in duplicate for each sample using SYBR Green Supermix (Bio-Rad) for 40 cycles with a 2-step program (5 s of denaturation at 96 °C and 10 s of annealing at 60 °C). Amplification specificity was assessed with a melting curve analysis. The relative expression of genes of interest was expressed relative to the expression of the reference gene, glyceraldehyde 3-phosphate dehydrogenase (GAPDH) or ribosomal protein L13a (Rpl13a). Analyses were performed with the Bio-Rad CFX manager 2.1-software (Marnes-la-Coquette, France). Primers were designed using Primer3 software, and sequences and their NCBI references were given in Table S1.

### 4.5. Ultrasound Imaging

Thermoregulated rats (*n* = 6 per group) were subjected to ultrasound measurements under 0.5% isoflurane anesthesia using an echocardiograph (ACUSSON S3000, Siemens, Erlangen, Germany) equipped with a 14.5-MHz linear transducer [6,8]. Arteries of interest were localized with 2D and color-Doppler ultrasound imaging on a horizontal cross-sectional view of the basis of the skull. The sample volume of the pulsed-Doppler was positioned to record and measure the spatial-averaged- time-averaged mean blood-flow velocities (mBFVs) in both intracranial internal carotid arteries (ICA) and basilar trunk (BT) before surgery, at the end of ischemia, at 15 min and one h after removal of the CCA occlusion. Heart rates were measured and reflected changes in cardiac output, as ventricular volume is quite invariable in newborns.

### 4.6. Measurement of Infarct Volume

Infarct size was determined on cresyl violet-stained sections on the animals subjected to US imaging (*n* = 6 per group), using an image analyzer (Image-pro, Paris, France). Infarct volumes were expressed as the percentage of the ipsilateral hemisphere.

### 4.7. Immunohistochemistry

Coronal 16-μm thick paraffin sections at the MCA level were stained for mouse anti-GFAP (G3893, 1:500 dilution; Sigma-Aldrich, Saint Louis, MO, USA) and goat anti-Iba1 (ab5076, 1:500; Abcam, Cambridge, UK) antibodies. For immunofluorescence, secondary antibodies coupled with the red fluorescent marker cyanine 3 (Jackson Immuno Research Laboratories, West grove, PA, USA) were used. GFAP density was done on 8-bit digital images collected using a high-resolution video camera (2560 × 1920 pixels) interfaced with a Nikon microscope under a 10× objective. Illumination and camera settings were maintained at the same level for image acquisition for all images. For each experimental group, two sections were imaged from 6 animals. Immunoreactivity signal (in the penumbra in the cortex) was segmented and counted using the Fiji distribution of ImageJ. Results are expressed in arbitrary units (A.U.).

### 4.8. Statistical Analysis

Data were expressed as mean ± SD. Mean blood-flow velocities (mBFV) were compared using repeated-measures ANOVA, and a post hoc Newman-Keuls test to analyze differences between two groups. One-way ANOVA with a post hoc Bonferroni test was used to analyze differences in gene expression between groups. The Kruskal-Wallis test was used to compare infarct volumes, Iba-1^+^ cells, and density of GFAP immunoreactivity between two groups. Statistical analysis of data was performed using PRISM 5 software (GraphPad, San Diego, CA, USA).

## Supplementary Materials

Supplementary materials can be found at http://www.mdpi.com/1422-0067/19/10/2995/s1.
