# Supplementary material for: Prostaglandin E1-Mediated Collateral Recruitment Is Delayed in a Neonatal Rat Stroke Model
Source: Int J Mol Sci. 2018 Sep 30;19(10):2995. doi: 10.3390/ijms19102995 (PMC6213314; doi:10.3390/ijms19102995)
Supplement: Supplementary file 1 [file ijms-19-02995-s001.pdf]

Online supplement

Prostaglandin E1-mediated collateral recruitment is delayed in a neonatal rat stroke model

Philippe Bonnin MD, PhD, Julien Pansiot Msc, Olivier Baud, Christiane Charriaut-Marlangue PhD

Supplemental Figure 1

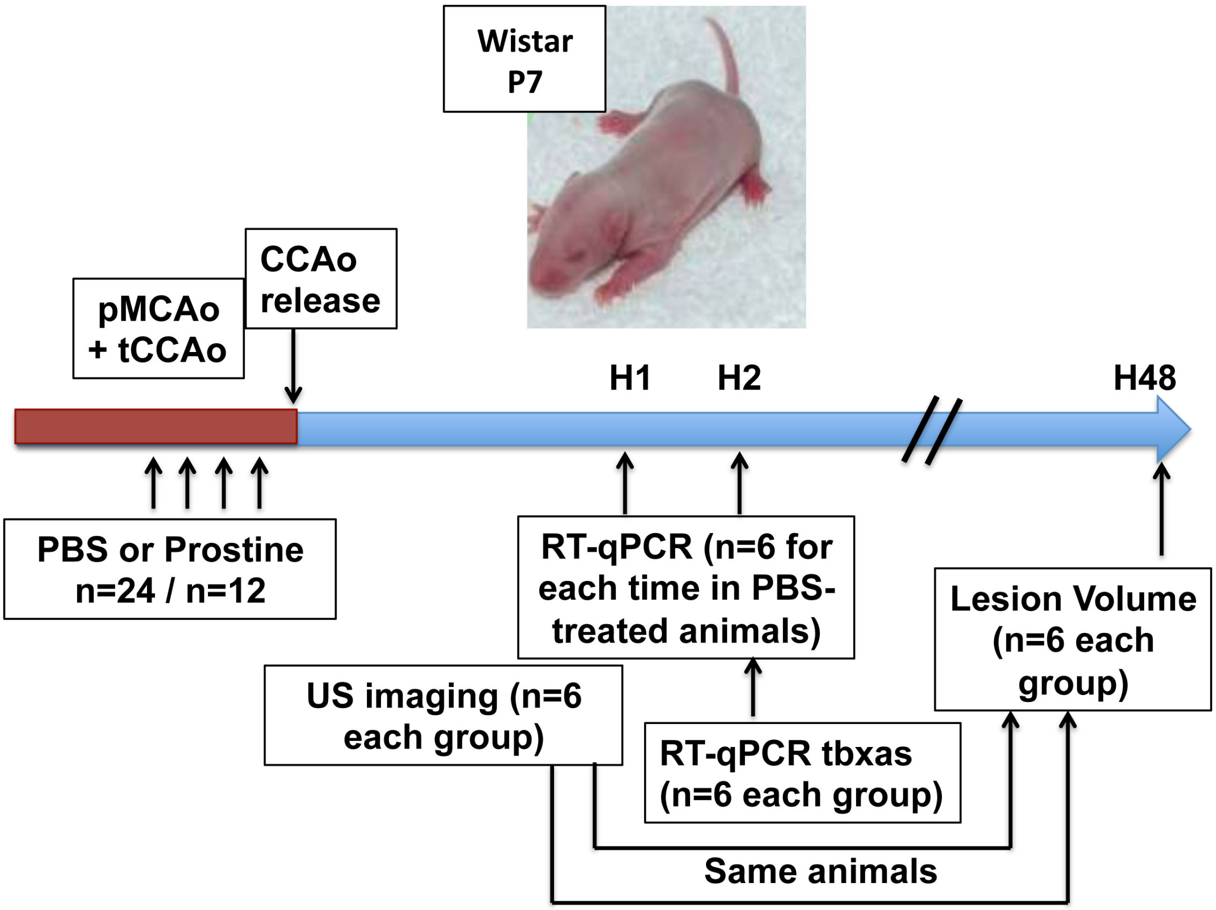

Outline of the experimental procedures for P7 Wistar rats subjected to ischemia-reperfusion (induced by pMCAo combined with a transient (50 min) and concomitant occlusion of both common carotid arteries – tCCAo). CCAo release indicates reflow in the carotid arteries. H1, H2 and H48 indicate that animals were sacrificed at 1, 2 and 48 hours after reflow.

Int. J. Mol. Sci. **2018**, *19*, 2995; doi:10.3390/ijms19102995

Supplemental Table 1

Gene	Alias	NCBI reference	Target protein and abbreviation		Sequence
**GAPDH**	GAPDH	NM_008084.2	Glyceraldehyde 3 phosphate dehydrogenase	GAPDH-rat-F	ggccttccgtgttcctac
				GAPDH -rat-R	tgtcatcatatctggcaggtt
**COX1**	Ptgs1	NM_017043.1	Rattus norvegicus prostaglandin-endoperoxide synthase 1 (Ptgs1), mRNA	cox1-rat-F	acctgcccctatgtctcctt
				cox1-rat-R	caaaactcctccctccagaa
**COX2**	Ptgs2	NM_017232.2	Rattus norvegicus prostaglandin-endoperoxide synthase 2 (Ptgs2), mRNA	cox2-rat-F	tcc tcc tgt ggc tga tga ct
				cox2-rat-R	cgg gat gaa ctc tct cct ca
**mPGES1**	Ptges1/Ptges	NM_021583.2	Rattus norvegicus prostaglandin E synthase (Ptges), mRNA	Ptges-rat-F	atccacttgacactgctcca
				Ptges-rat-R	atccacttgacactgctcca
**mPGES2**	Ptges2	NM_001107832.1	Rattus norvegicus prostaglandin E synthase 2 (Ptges2), mRNA	Ptges2-rat-F	gcagatgtatggcggaaagg
				Ptges2-rat-R	gggagagatgagatgcacca
**PGIS**	Ptgis	NM_031557.2	Rattus norvegicus prostaglandin I2 (prostacyclin) synthase (Ptgis), mRNA	Ptgis-rat-F	gtgcctggggaagagctatg
				Ptgis-rat-R	gtcaaactccgggacctctg
**TxS**	Tbxas1	NM_012687.1	Rattus norvegicus thromboxane A synthase 1 (Tbxas1), mRNA	Tbxas1-rat-F	gttggggcttctcaagttcg
				Tbxas1-rat-R	tgagaacgctgatgtggagt
**Rpl13a**	Rpl13a	NM_009438.5	Ribosomal protein L13a	rRpl13-F	tccgaagaagggagacagtt
				rRpl13-R	cttctcctcttccgtgatgg

Supplemental Figure 2

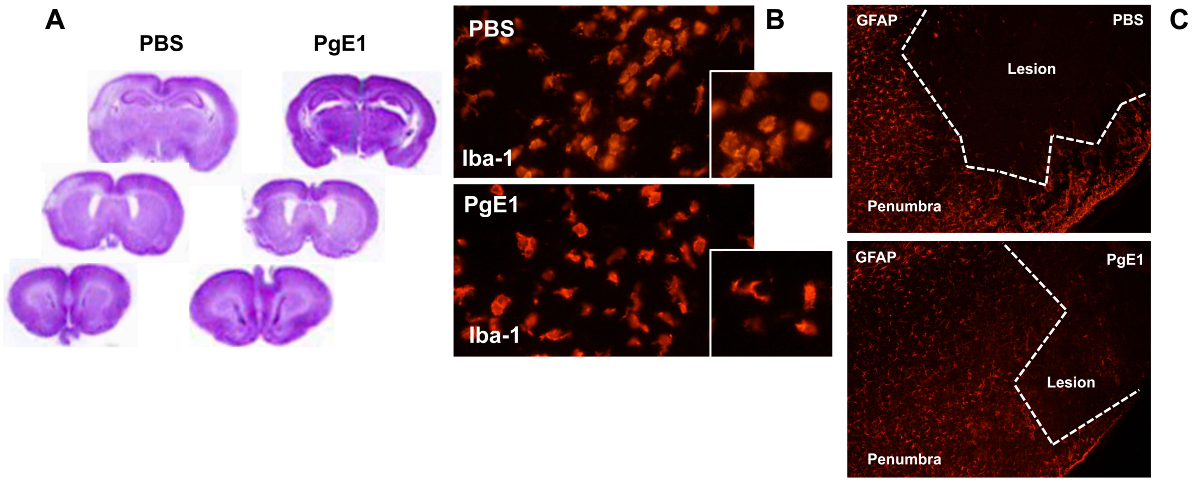

PgE1 improves better outcomes in neonatal ischemia. **A**: Representative cresyl violet-stained sections in a PBS- (left) and PgE1-treated (right) animal sacrificed at 48 hours after ischemia. Note a smaller pale lesion in sections from the PgE1-treated animal. **B-C:** Representative microglial (Iba-1) and astrocyte (GFAP) immunoreactivity in a PBS- and PgE1-treated animal at 48 hours. Note that the GFAP density is reduced in PgE1-tretaed animals.
